# High-Dynamic-Range Spectral Reflectance for the Segmentation of Paint Pigment: Application to Dalí’s Oil Painting Dos Figuras (1926)

**DOI:** 10.3390/s23094316

**Published:** 2023-04-27

**Authors:** Antonio Alvarez Fernandez-Balbuena, Angela Gómez-Manzanares, Juan Carlos Martínez Antón, Jorge García Gómez-Tejedor, Santiago Mayorga-Pinilla, Humberto Durán Roque, Daniel Vázquez Moliní

**Affiliations:** 1 Department of Optics, University Complutense of Madrid, 28040 Madrid, Spain; antonioa@ucm.es (A.A.F.-B.);; 2 Museo Nacional Centro de Arte Reina Sofía, 28012 Madrid, Spain; 3 Hdurán: Imágenes para Estudios Técnicos Conservación y Restauración, 28005 Madrid, Spain

**Keywords:** multispectral imaging, cultural heritage, pigment segmentation, spectral reflectance, high-dynamic-range

## Abstract

Restorers and curators in museums sometimes find it difficult to accurately segment areas of paintings that have been contaminated with other pigments or areas that need to be restored, and work on the painting needs to be carried out with minimum possible damage. It is therefore necessary to develop measurement systems and methods that facilitate this task in the least invasive way possible. The aim of this study was to obtain high-dynamic-range (HDR) spectral reflectance and spatial resolution for Dalí’s painting entitled Two Figures (1926) in order to segment a small area of black and white pigment that was affected by the contact transfer of reddish pigment from another painting. Using Hypermatrixcam to measure the HDR spectral reflectance developed by this research team, an HDR multispectral cube of 12 images was obtained for the band 470–690 nm in steps of 20 nm. With the values obtained for the spectral reflectance of the HDR cube, the colour of the area of paint affected by the transfer was studied by calculating the a*b* components with the CIELab system. These a*b* values were then used to define two methods of segmenting the exact areas in which there was a transfer of reddish pigment. The area studied in the painting was originally black, and the contamination with reddish pigment occupied 13.87% to 32% of the total area depending on the selected method. These different solutions can be explained because the lower limit is segmentation based on pure pigment and the upper limit considers red as an exclusion of non-black pigment. Over- and under-segmentation is a common problem described in the literature related to pigment selection. In this application case, as red pigment is not original and should be removed, curators will choose the method that selects the highest red area.

## 1. Introduction

Restoration and curation techniques in the area of cultural heritage require the use of technologies that allow for the characterisation of the pigments that make up artworks in a precise and non-invasive manner [1,2]. To carry out this characterisation, colorimetric analysis based on spectral reflectance measurements is used [1,3]. The spectral reflectance parameter of an artwork can be used in several ways—for example, to characterise pigments [4], to verify the presence of a pigment in different parts of the artwork through pigment segmentation [5], and to control and monitor the deterioration of the artwork over time, among many other applications.

The proper characterisation and identification of pigments in artworks depend on the spatial and spectral resolution of the system [6]. Imaging spectrophotometers have generally been used to obtain spectral reflectance for artworks via a non-contact process [7,8]. However, spectrophotometers have a limited spot measurement area and are therefore unsuitable for use with large artworks [9,10]. To eliminate this spatial limitation, multispectral imaging (MSI) and hyperspectral imaging (HSI) are used to obtain diffuse spectral reflectance for artworks [5,11,12].

MSI and HSI can obtain diffuse spectral reflectance with a resolution that depends on the pixel size of the system sensors and the optical quality of the system, as they measure the spectral information of each pixel (px) of the image [13]. Commercially available MSI and HSI capture systems provide imaging with high efficiency; however, there is a tradeoff between the two methods, as hyperspectral cameras can obtain images with a high spectral resolution but low spatial resolution, while multispectral cameras can obtain images with a high spatial resolution but low spectral resolution [14]. For example, the Specim IQ is a hyperspectral camera that is capable of imaging in 204 spectral bands, covering the spectral range 400–1000 nm, with a spatial resolution of 1260 × 960 px, whereas the Micasense ReddishEdge-P multispectral camera images in five spectral bands, with a spatial resolution of 2464 × 2056 px. The precise spatial and spectral resolution that defines the difference between the two methods is unclear. However, recent research explains that the difference lies in the continuum of the detected spectrum. Hyperspectral imaging systems are characterised by the fact that they are able to image over a continuous range of the spectrum [15,16].

Numerous studies have been carried out with the aim of capturing HSI with a high spatial resolution. In their research, Yifan et al. developed an algorithm that could obtain images with the spatial resolution of MSI and the spectral resolution of HSI, using the 3D wavelet transform [17]. In order to carry out HSI with high spatial resolution, Akhtar et al. developed an algorithm that combined an RGB image with higher spatial resolution to spectrally define the RGB channels of the image [18].

In the field of cultural heritage restoration and conservation, it is necessary to obtain HSI with high spatial resolution to perform the segmentation of large artworks. Super-resolution algorithms are widely used to perform pigment segmentation of artworks [2,19]. The aim of super-resolution segmentation is to obtain images with high-spatial-resolution information from images with a low spatial resolution, based on a prior grouping of pixels with the same spectral characteristics [5,19,20].

When documenting and analysing artworks, the gigapixel technique is widely used with RGB cameras, as commercial RGB cameras enable images with a high spatial resolution to be obtained [21].

Pigment segmentation is a technique that can be mathematically executed with the HSI technique and post-processing. Post-processing is used in segmentation because over- or under-segmentation can take place [2]. Magro et al. [2] reported that HSI reduced 43% over-segmentation in the proposed algorithm. Different pigments present different reflection characteristics, so in the visible spectrum, we can use this information to identify pigments and normalise the spectral reflectance difference to a database that can identify pigments. This method was used by Li J. et al. [5] to characterise and segment the pigments in the Mogao Caves in Dunhuang.

However, the colorimetric properties of the pigments that make up the paints alter over time due to the action of temperature, humidity, and exposure to light, among other things [22]. In addition, the pigments in the paints combine with each other, which alters their colour properties and results in variations in spectral reflectance, making characterisation difficult to carry out in the segmentation of these pigments [23]. Hence, to properly characterise and segment the pigments in an artwork, it is necessary to implement techniques and algorithms that consider these possible mixtures of pigments, in addition to using systems with a high spatial resolution.

A Spectral Angle Mapper (SAM) is a technique that is often used to identify similarities between the spectral profile of pigments in a painting and reference pigments that have been previously characterised and are available in a library [24,25]. In their study, Deborah et al. performed pigment segmentation of Edvard Munch’s painting The Scream using an algorithm based on spectral divergence [26].

In this work, we present spectral reflectance measurements of Dalí’s painting using multispectral images with high-dynamic-range and 4K spatial resolution. Based on these measurements, two methods are developed for performing pigment segmentation and identification, taking into account variations due to pigment mixing.

Section 2 of this article describes the materials and methods, including the MSI system used, and explains the measurement and calibration procedures. Section 3 presents the results, which demonstrate the high dynamic range of the spectral reflectance of the artwork, and introduces the methods used to carry out pigment segmentation. Finally, Section 4 and Section 5 present a discussion and the conclusions reached after carrying out this study.

## 2. Materials and Methods

**Figure 1:**
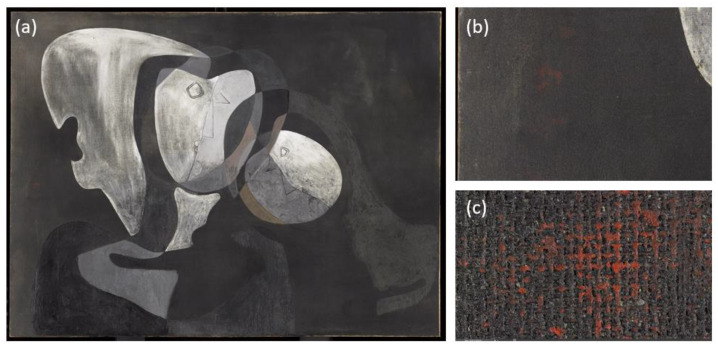
(**a**) High-spatial-resolution RGB image of Dalí’s painting Two Figures (1926), provided by the Museo Nacional Centro de Arte Reina Sofía; (**b**) enlarged section of an area of the painting; (**c**) enlargement of one of the areas affected by the reddish pigment (image dimensions 6.8 × 4.9 cm).

Salvador Dalí’s Two Figures (1926) was affected by the transfer of a reddish pigment when another painting came into contact with this work. Figure 1b,c show the detail of the reddish pigment, obtained from a gigapixel image taken by the restoration team of the Museo Nacional Centro de Arte Reina Sofía (Figure 1a).

### 2.1. Multispectral Image Capture System

The segmentation and localisation of the area of the artwork affected by the reddish pigment require previous colorimetric characterisation based on the spectral reflectance. In order to identify this pigment, this research team has developed a multispectral image capture system (Hypermatrixcam) that is capable of obtaining spectral images with a high spatial resolution [27].

**Figure 2:**
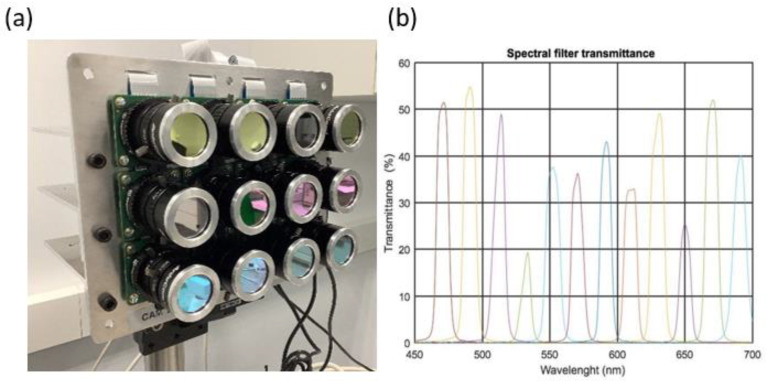
(**a**): Front of Hypermatrixcam; (**b**): transmittance curves for the spectral filters, ordered from left to right as 470, 490, 510, 530, 550, 570, 590, 610, 630, 650, 670, and 690 nm.

This image capture system consists of a matrix array of 12 RGB sensors with 4K spatial resolution (Figure 2a); patent ES2819052-B2. A spectral bandpass filter covering the visible spectrum range of 470–690 nm is placed on each of the sensors (Figure 2b).

### 2.2. Experimental Setup

In its recommendation 15-2004, the International Commission on Illumination (CIE) established specific geometrical conditions for spectral reflectance measurements depending on the surface to be tested and the instrument used [28]. Furthermore, Fairchild, in his book, recommends that for reflectance measurements of artworks using spectrophotometers or multispectral or hyperspectral cameras, the light source should be placed at an angle of 45° to the sample to avoid direct reflections [29].

Dalí’s painting Two Figures (1926) measures 1.98 × 1.49 m, and the distance at which the camera should be placed with respect to the painting depends on the field of view of the measurement system. Each sensor has a field of view of 30° × 27.7° (horizontal × vertical); hence, considering the geometric displacement of the 12 sensors, the field of view of Hypermatrixcam is 23.4° × 20.5° (horizontal × vertical). This gives a value of 4.8 m for the distance $d_{Dali}$ necessary to obtain multispectral images of the entire painting while leaving a margin for colorimetric patterns.

**Figure 3:**
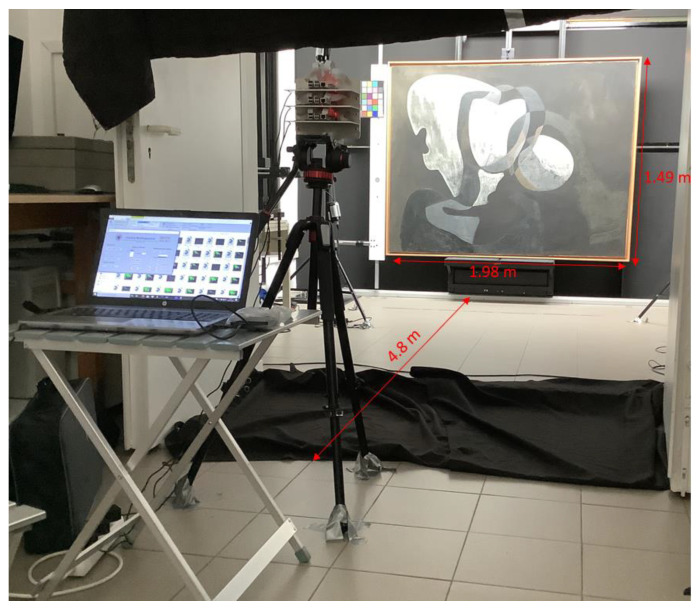
Geometric arrangement and distance used during the spectral reflectance measurements of Dalí’s painting at the Museo Nacional Centro de Arte Reina Sofía.

This distance for the measurement system allows multispectral images of the painting to be obtained with a resolution of 3322 × 2510 pixels (horizontal × vertical). The expression $rh_{Dali}=S_{Dali}/HSI_{Dali}=0.59\;\mathrm{mm}$ was used, where $S_{Dali}$ is the size of the painting in mm and $HSI_{Dali}$ is the size of the image obtained in pixels. This made it possible to record areas of 0.35 mm^2^ of the artwork for each pixel of the HSI obtained (Figure 3).

#### 2.2.1. Lighting System

Three Art Lux 500 Flex LED array panels with a total size of 410 × 1830 mm (horizontal × vertical) were chosen as the light source for the reflectance measurements. The choice of LED panels was made due to the absence of UV and IR radiation in their emission to avoid damaging the artwork [30]. An LED array was placed at a vertical angle of 45° to the centre of the painting in order to ensure maximum uniformity in the illumination of the painting.

#### 2.2.2. System Calibration

Once the geometrical configuration for the spectral reflectance measurements had been established, the system was calibrated. For this purpose, an HSI image was initially captured without the painting; as a calibration standard, we used a foamed PVC sheet, a uniform material with good diffusive properties and a uniform reflectance value. The PVC sheet had to be of the same dimensions as the Dalí painting in order to calibrate the nonuniformity of the illumination system. The mean reflectance $\rho_{white}\left(\lambda \right)$ of this material was measured with a PR-655 spectrophotometer (Photo Research Inc., Topanga, CA, USA) in the geometric configuration 0–45° directional [28]. Measurements were performed at 18 different equidistant points, and a value of $\rho_{white}\left(\lambda \right)=85.07\%\pm 2.68\%$ was obtained.

An image of the PVC was then captured with Hypermatrixcam to give the spectral HDR of the reference target $I_{HDR_{white}}{\left(\lambda \right)}_{\left(n,m\right)}$, where (*n*, *m*) is the size in pixels of the images taken. With the resolution of Hypermatrixcam, these values were *m* = 3322 pixels and *n* = 2510 pixels.

Without changing the positions of the camera or the lighting system, images of Dalí’s painting were then captured in the same place where the foamed PVC was previously located to give the spectral HDR of the painting $I_{HDR_{s}}{\left(\lambda \right)}_{\left(n,m\right)}$.

To complete the measurement, multispectral images of the dark current were captured, with the sensors covered, to give the HDR of the dark current of the system $I_{HDR_{dark}}{\left(\lambda \right)}_{\left(n,m\right)}.$

### 2.3. HDR Image Capture

Multispectral and hyperspectral cameras typically consist of sensors with a limited dynamic range, resulting in low-dynamic-range (LDR) images [31]. These LDR images will contain saturated and/or underexposed pixels for a given exposure time. In addition, the response of these sensors is not linear and shows areas of nonlinearity for low and high values of the grey levels.

In order to obtain spectral reflectance measurements using HSI, all of the pixels must have a value for the grey level that is within the linearity zone of the sensor [32]; otherwise, the spectral characterisation will be altered.

**Figure 4:**
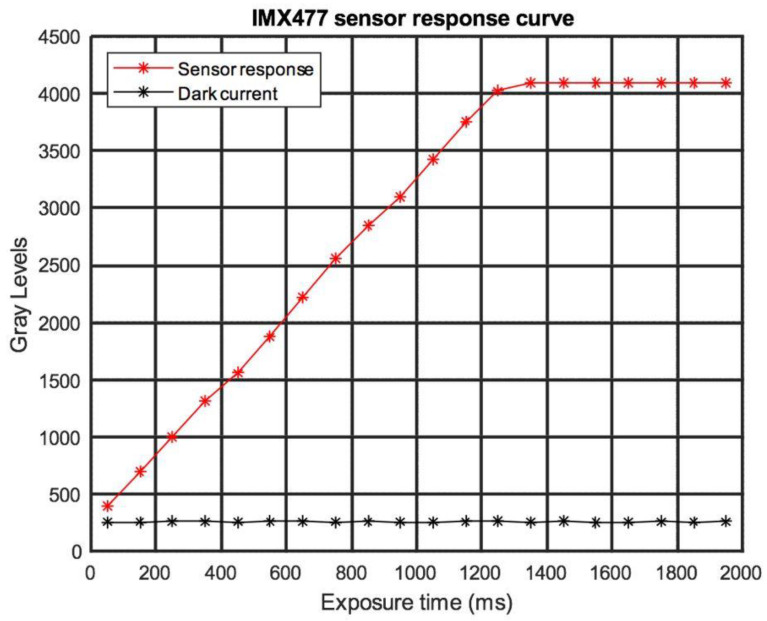
Experimental response curve for the IMX477 sensor.

To define the linearity limits in the response of the CMOS sensors, the curve of the sensors as a function of exposure time was obtained (Figure 4). For these measurements, white LED illumination was used, without the superimposition of spectral filters on the sensors, since the distortion between the response curves of the sensors for the different wavelengths is minimal in the visible spectrum [33].

**Figure 5:**
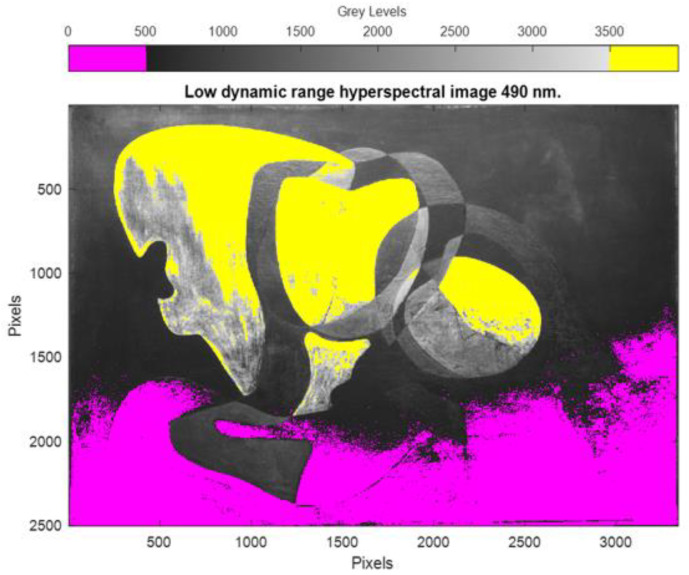
LDR multispectral image of Two Figures (1926) at 490 nm, with underexposed (yellow) and overexposed (pink) pixels.

Dalí’s painting Two Figures (1926) has very light and dark areas on its surface. As shown in Figure 5, capturing images at a certain exposure time results in pixels that are outside the linearity zone of the sensor, with both saturated (yellow) and underexposed (pink) pixels in the same image.

The system used in this work allowed HDR multispectral images to be obtained by capturing them over different exposure times. Based on LDR images, an HDR image is obtained from a combination of the grey levels in each pixel that are not saturated or underexposed.

The MSI acquisition system consisted of RGB sensors and spectral filters. The sensitivity of the RGB sensors and the transmittance of the spectral filters varied spectrally for each wavelength, and images were therefore captured with different exposure times. These exposure times were selected by adjusting them to minimise the saturated pixel values in each of the intervals of the working spectrum.

The range of exposure times chosen for wavelengths in the range 470–630 nm was 0.5–12.5 s in steps of 3 s. For wavelengths in the range 650–690 nm, the range of exposure times was 8–20 s in steps of 4 s. After obtaining the HDR multispectral images of the calibration, paint, and dark current, the high-dynamic-range spectral reflectance $\rho_{s}{\left(\lambda \right)}_{\left(n,m\right)}$ at each pixel (*n*, *m*) was calculated using Equation (1):

(1) $$\rho_{s}{\left(\lambda \right)}_{\left(n,m\right)}=\rho_{white}{\left(\lambda \right)}_{\left(n,m\right)}\frac{I_{HDR_{s}}{\left(\lambda \right)}_{\left(n,m\right)}-I_{HDR_{dark}}{\left(\lambda \right)}_{\left(n,m\right)}}{I_{HDR_{white}}{\left(\lambda \right)}_{\left(n,m\right)}-I_{HDR_{dark}}{\left(\lambda \right)}_{\left(n,m\right)}}$$

where $\rho_{white}{\left(\lambda \right)}_{\left(n,m\right)}=85.4\%$ is the mean spectral reflectance of the PVC used for calibration; $I_{HDR_{white}}{\left(\lambda \right)}_{\left(n,m\right)}$ is the MSI of the PVC used as calibration material; $I_{HDR_{s}}{\left(\lambda \right)}_{\left(n,m\right)}$ is the MSI of Dali’s painting; and $I_{HDR_{dark}}{\left(\lambda \right)}_{\left(n,m\right)}$ is the HSI of the dark current.

### 2.4. Basis of Methods for Pigment Segmentation

A spectral reflectance cube can be used for pigment segmentation; in this case, two methods are described and applied to the painting. Both methods require processing a spectral reflectance cube with a standard illuminant to obtain CIELab data, a* and b*. These data are related to the pixel colour.

Due to the mixture between pigments and the alterations they suffer in terms of their colorimetric properties over time, the segmentation of two pigments whose reflectance spectra are close to each other is difficult [23]. When processing the reflectance spectra according to a* and b* values, this division suffers the same problem, and establishing a separation boundary becomes quite arbitrary. To help in the segmentation process, we need some extra information. We propose two methods that basically focus on image areas with a predominance of a type of pigment (black or reddish in our application case).

Method 1: Inverse segmentation by characterising a black area without the transfer of reddish pigment.

In this method, additional data consist of obtaining representative a* and b* data of the black pigment from an area that has no red pigment at all.

If you segment black according to these data, then red will be the remaining pixels because there are only two colours in the selected area.

In this method, we can define by a set *U* with all a*b* data in the image:

(2) $$\;\;U=\left\{\;a^{*}b^{*}\right\}\;$$

Additionally, we can define a subset *K*:

(3) $$K=\left\{a*b*\;|\;black\;pigment\right\},\;\mathrm{with}\;K\subseteq U$$

where K is a subset containing CIELab black pigment that was selected using an area of the image with only black pigment. In practice, this defines a closed boundary βb in the a*b* data representation that permits a simple segmentation. This process can be described by

(4) $$U\backslash K=R_{M1}$$

where $R_{M1}$ is the subset of data that are non-black. So, in a bi-coloured image, red and black, this is red for a different saturation level.

Method 2: Segmentation based on an area with a high incidence of red pigment.

This method takes a* and b* data from a selected area strongly related to the red pigment. Selection in other areas is made by obtaining data that have the a* and b* that are inside the boundary obtained by directly analysing the red pigment.

In this case, any image subset that only has red pigment gives:

(5) $$R_{M2}=\{a^{*}b^{*}|\;red\;pigment\}$$

In Equation (5), the $R_{M2}$ set is obtained by selecting a point cloud area with only red pigment; then, the a*b* data are surrounded by a boundary β_red_ that will enclose all red pigment related to the selected boundary. So, it is possible to segment, in any image with any colour, with these set data to obtain red.

## 3. Results

### 3.1. High-Dynamic-Range Spectral Reflectance

**Figure 6:**
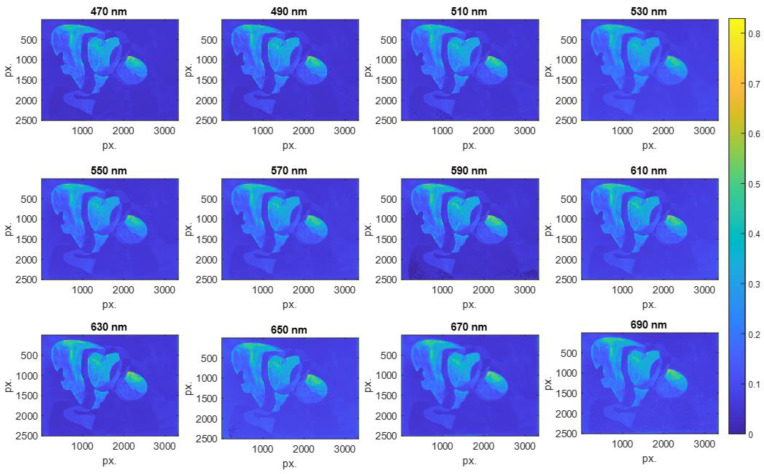
HDR spectral reflectance obtained from Dalí’s painting Two Figures (1926) in the spectral range 470–690 nm in steps of 20 nm.

Multispectral images (MSI) were acquired with different exposure times to give HDR images of Dalí’s painting Two Figures (1926). From these images, and using Equation (1), the HDR spectral reflectance was processed, as shown in Figure 6. The spectral resolution of the measurements was 470–690 nm in steps of 20 nm, and the spatial resolution was 3322 × 2510 px.

### 3.2. Pigment Segmentation

The segmentation of the pigments in the painting was carried out using the multispectral images obtained as described above. Each multispectral image had a spatial resolution of $sr_{HDR}=3322\times 2510\;\mathrm{px}=8.3\;\mathrm{Mpx}$ and a spectral resolution of 12 channels.

**Figure 7:**
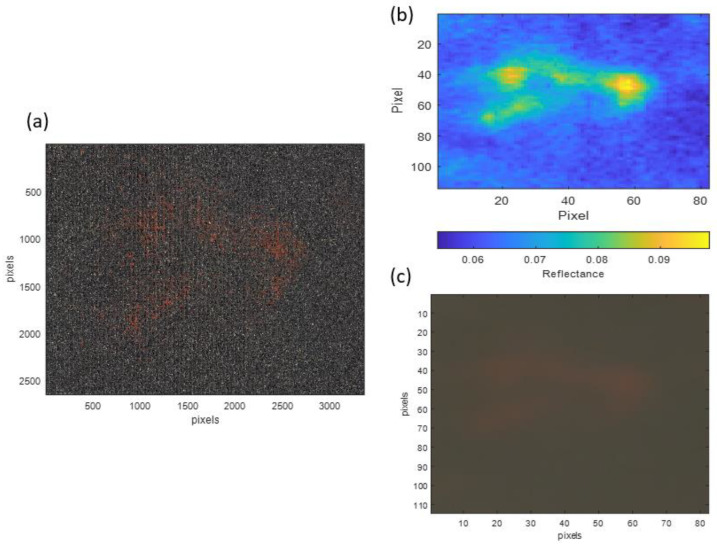
Images of Reference Area 1 used for analysis: (**a**) high-spatial-resolution RGB image; (**b**) image with 4K HDR spectral reflectance information at 630 nm; and (**c**) RGB image obtained from HDR spectral reflectance measurements.

To segment the pigment, it was first necessary to characterise it colorimetrically. For this purpose, a small area of the paint affected by the transfer of the reddish pigment (Reference Area 1) was selected, with a size of 6.8 × 4.9 cm. Figure 7a shows a high-spatial-resolution gigapixel RGB image corresponding to Reference Area 1, while Figure 7b shows the HDR spectral reflectance value for the same area at a wavelength of 630 nm, where the reddish pigment is detected as an area of pixels with higher reflectance (shown in yellow on the false colour map). Figure 7c shows the RGB image obtained from the HDR spectral reflectance measurements.

Using illuminant A as a reference and a standard CIE 1931 observer, the first step is to calculate the XYZ tristimulus values from the reflectance spectrum of each pixel of Reference Area 1, $\left(n,m\right)$, $\rho_{s}{\left(\lambda \right)}_{\left(n,m\right)}$.

The CIELab $a^{*}b^{*}$ colour values of the selected area $\left(n,m\right)$ are calculated from the tristimulus data $XYZ_{\left(n,m\right)}$ [29,34].

**Figure 8:**
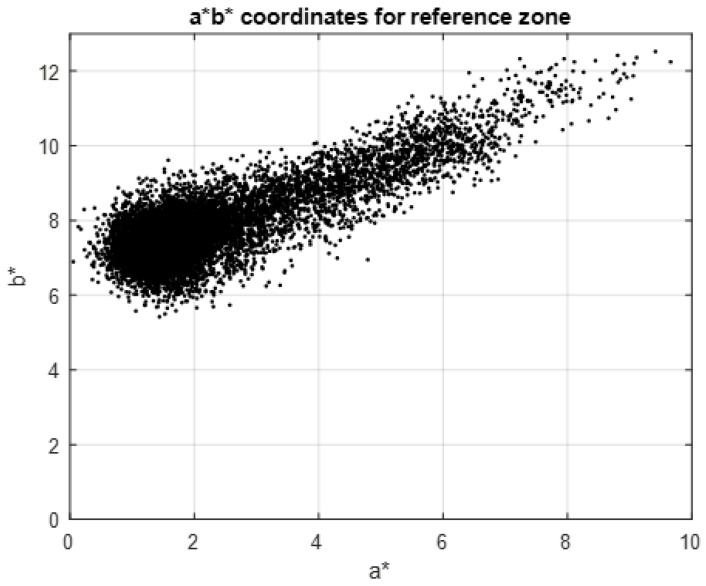
Graphical representation of the CIELab a*b* values for the reddish pigment and the black pigment in Reference Area 1.

Figure 8 shows the data for $a^{*}{}_{\left(n,m\right)},\;b^{*}{}_{\left(n,m\right)}$. The CIELab enhancement in $a^{*}$ is standard for the red colour enhancement, and the hue angle following the enhancement in the components $a^{*}{}_{\left(n,m\right)},\;b^{*}{}_{\left(n,m\right)}$ is observed, but it is not easy to find a delimiter between the reddish and black pigment. Two methods are proposed below for carrying out this segmentation.

**Method 1: Inverse segmentation by characterising a black area without the transfer of reddish pigment**

**Figure 9:**
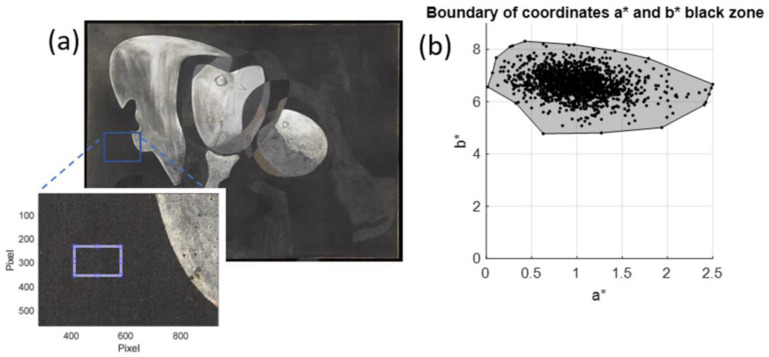
(**a**) Black area of the image selected for analysis; (**b**) $a^{*}b^{*}{}_{\left(black\right)}$ values for this area, bounded by an envelope.

In this case, a black area was selected (Figure 9a), and its components $a^{*}b^{*}{}_{\left(black\right)}$ were calculated. The components $a^{*}b^{*}{}_{\left(black\right)}$ formed a point cloud, which was plotted next to the boundary of the $a^{*}b^{*}{}_{\left(black\right)}$ values; this boundary allowed us to segment the rest of the $a^{*}b^{*}$ values present in the painting (Figure 9b).

**Figure 10:**
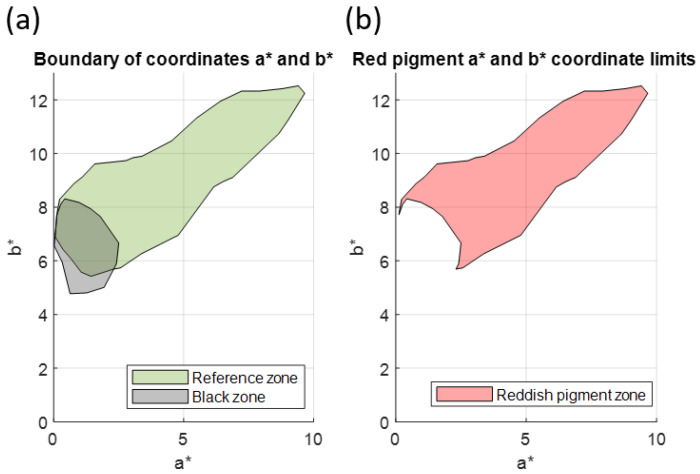
(**a**) Graphical representation of the bounded regions of the values $a^{*}b^{*}{}_{\left(black\right)}$ and the values $a^{*}{}_{\left(n,m\right)},\;b^{*}{}_{\left(n,m\right)}$; (**b**) graphical representation of the bounded region of the values $a^{*}b^{*}{}_{reddish\_1}$.

Once the region with the values $a^{*}b^{*}{}_{\left(black\right)}$ had been defined, it was represented next to the envelope bounding the values $a^{*}{}_{\left(n,m\right)},\;b^{*}{}_{\left(n,m\right)}$ of Reference Area 1, which was selected to characterise the red pigment (Figure 10a). The geometric difference between the two regions was then found in order to define the region with the $a^{*}b^{*}{}_{reddish\_1}$ values corresponding to the reddish pigment (Figure 10b).

**Figure 11:**
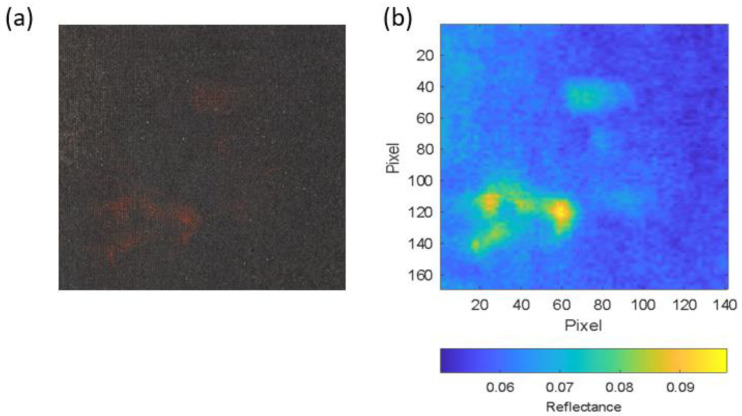
(**a**) RGB image segmentation area; (**b**) reflectance image segmentation area at 630 nm.

To validate this method, we selected an area (called the segmentation area) that was larger than the one selected as a reference for the analysis. Figure 11a shows the segmentation area in an RGB image, and Figure 11b shows the segmentation area in a false colour image, in which the reflectance at 630 nm is represented.

**Figure 12:**
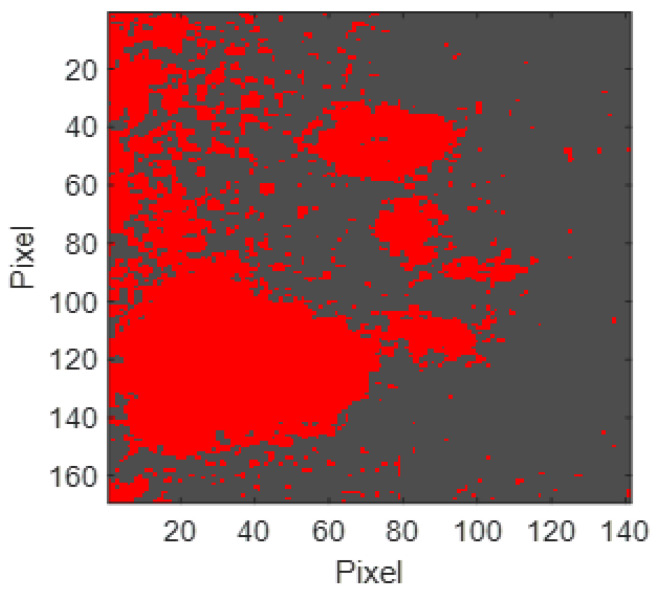
Segmentation of the reddish pigment in the segmentation area from the $a^{*}b^{*}{}_{reddish\_1}$ values (reddish pixels represented in false red).

Using the $a^{*}b^{*}{}_{reddish\_1}$ values obtained above, the reddish pigment present in the segmentation area was segmented (Figure 12).

**Method 2: Segmentation based on an area with a high incidence of red pigment**

**Figure 13:**
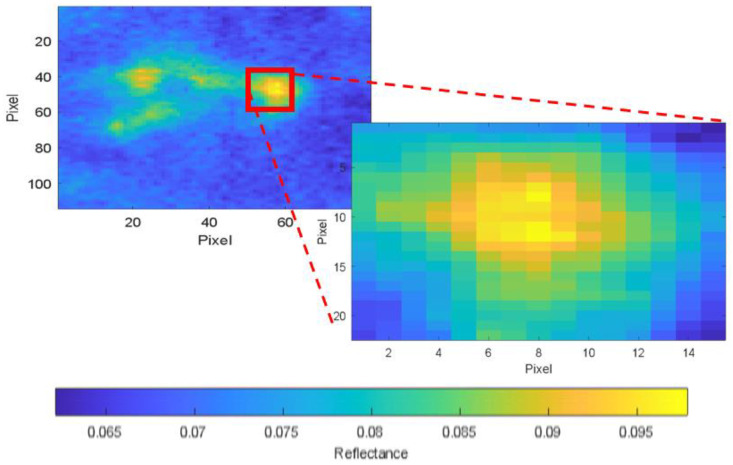
Image of Reference Area 2 with HDR spectral reflectance information at 630 nm.

Rather than performing segmentation of the reddish pigment based on the previous characterisation of a black area, it is also possible to perform segmentation based on the HDR spectral reflectance of an area with only a high incidence of red pigment, called Reference Area 2 (Figure 13).

**Figure 14:**
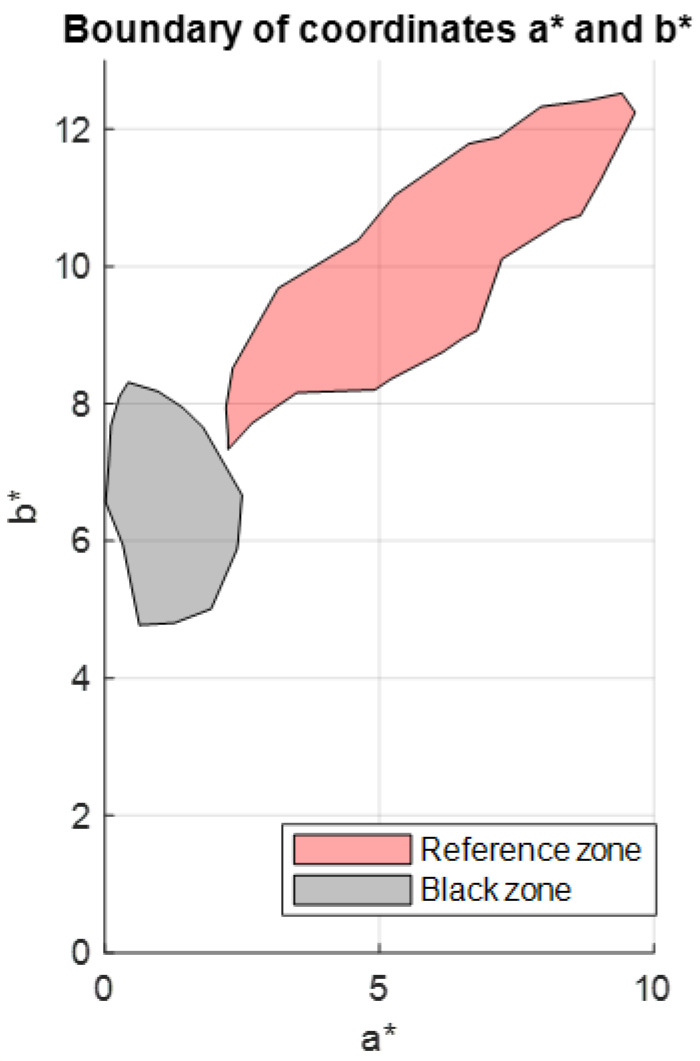
Graphical representation of bounded regions with the values $a^{*}b^{*}{}_{\left(black\right)}$ and with the values $a^{*}b^{*}{}_{reddish\_2}$.

In the same way as described in the previous section, the coordinates $a^{*}b^{*}{}_{reddish\_2}$ for Reference Area 2 were obtained using illuminant A and a standard CIE 1931 observer at 2° as the reference illuminant. The region bounding the $a^{*}b^{*}{}_{reddish\_2}$ values was then obtained and plotted next to the region of $a^{*}b^{*}{}_{\left(black\right)}$ values (Figure 14).

**Figure 15:**
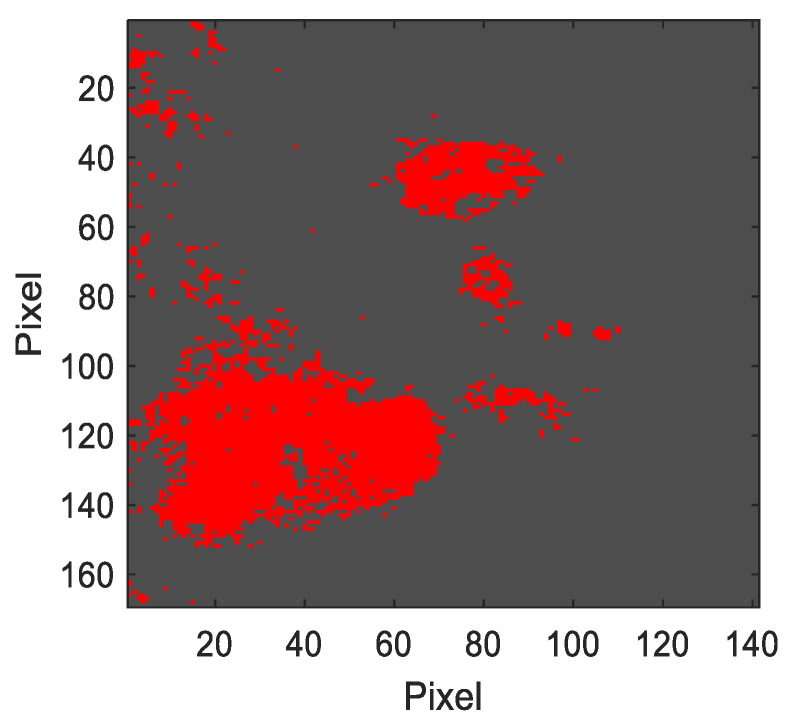
Segmentation of the reddish pigment in the segmentation area from the $a^{*}b^{*}{}_{reddish\_2}$ values (reddish pixels represented in false red).

Since these two regions do not overlap, segmentation can be performed directly on the segmentation area based on the region with the values $a^{*}b^{*}{}_{reddish\_2}$ (Figure 15).

### 3.3. Comparison of Proposed Methods

The results obtained with both methods are shown in Table 1. Using Method 1, 4307 more pixels were obtained compared to those obtained with Method 2, equivalent to 18.07% of the affected segmentation area.

**Figure 16:**
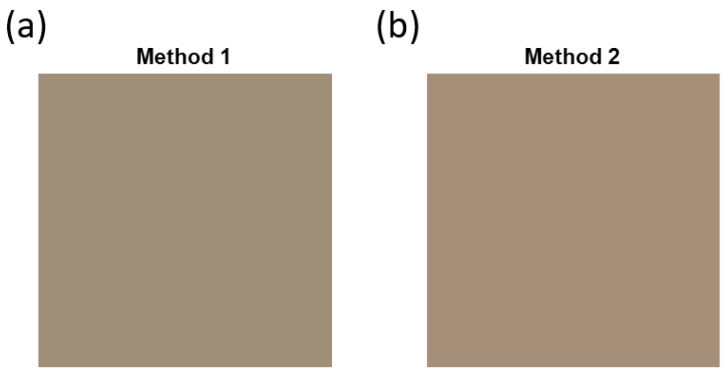
RGB patch obtained through the mean value of the spectral reflectance of the pixels included in the segmentation using (**a**) method 1 and (**b**) method 2.

For each of the two proposed methods, the mean value of the spectral reflectance spectra of all pixels included in the segmentation was calculated. The spectral reflectance values for each method have been processed to obtain the corresponding RGB values for method 1 (Figure 16a) and method 2 (Figure 16b). The CIE 1931 2° Observer Standard and Illuminant A were used for the processing. The RGB values have been multiplied by a factor of two to increase their luminance level and improve visual colour appreciation.

## 4. Discussion

This study presents measurements of the HDR spectral reflectance for Dalí’s painting entitled Two Figures (1926) and a colorimetric analysis of the reddish pigment that affects the work due to the transfer between pigments.

To enable non-contact measurements, the segmentation and quantification of the pigments that make up a painting are typically carried out through a colorimetric analysis using spectral reflectance measurements. Since pigments are distributed in very small quantities over the artwork, in order to carry out characterisation and segmentation, it is necessary to use methods that allow spectral information to be obtained with high spectral and spatial resolution. Commercial MSI and HSI capture systems do not have sufficient spatial resolution to segment pigments in large artworks. Hence, although a combination of HSI and MSI increases the spatial resolution of HSI, it is not sufficient to perform pigment segmentation accurately [17]. Adequate spatial resolution is therefore achieved by fusing HSI with RGB images [18].

Super-resolution algorithms perform a posteriori processing of the spectral reflectance information obtained from HSI [2,19], and the information obtained in this way allows for segmentation and subsequent mapping of the different pigments used in the artwork. However, these algorithms are not able to detect small amounts of pigment in paintings or sculptures.

With the multispectral image capture system used in this research, it was possible to obtain spectral information of an area of 0.35 mm^2^ of Dalí’s painting in each pixel of the image obtained. The spectral and spatial resolution of these HDR spectral reflectance measurements allowed us to characterise the reddish pigment present in the painting.

Due to the difficulty of defining the thresholds between a pure pigment, its mixtures, and the rest of the pigments, SAM and divergence techniques are interesting for use in pigment segmentation [24,25,26]. When a spectral library is taken as a reference, they allow us to quantify the similarity between a pure pigment and its mixtures.

In this study, the pigment present in the paint itself was used for characterisation, and a comparison with reference spectral libraries was avoided. Furthermore, prior to segmentation, the two methods proposed here were used to characterise the pigment in its pure and mixed states based on the a* and b* coordinates, thus simplifying the process.

In Method 1, a segmentation was performed that included areas in which the pigment had been mixed and which were imperceptible to the naked eye. Method 2 involved an analysis based on the most visible areas, since Reference Area 2 was an area of the artwork containing pure red pigment. The choice between the methods described above will depend on the needs of restoration and conservation experts in the field of cultural heritage, since the line that delimits a pigment is not clear; this is especially the case if red and black pigments are compared, as was the case here, as they are colorimetrically similar to each other. As pigment is on the painting surface, the reflectance is not always strictly related to the added pigment; this is because is the deposition layer is thin, and the reflectance is a mix between the original layer and the added pigment layer. In this way, pigment segmentation has a limitation in method 2 because this selection only obtains a big pigment layer. Method 1 can select the thinnest layer of red because it is obtained by the exclusion of black; this is only useful if there is only black and red. If there are additional colours, they will be segmented like red because they are not black.

In the application case of this Dalí painting, the curator selection of interest is the highest area (Method 1); thus, all of it is intrusive pigment that must be eliminated because it is a transfer from another painting.

## 5. Conclusions

This study has presented HDR spectral reflectance measurements of Dalí’s painting entitled Two Figures (1926), with 12 spectral bands and high spatial resolution. The data obtained represent objective information on the colours of the painting for future interventions and analyses of its state of conservation. Our approach allows for a resolution of 3322 × 2510 px. As part of our analysis of the spectral reflectance of the painting, the reddish pigment transfer affecting a piece of the artwork was characterised and segmented with two proposed methods. In the same area, Method 1 gives 32% of reddish pigment and method 2 gives 13.87% of reddish pigment. Method 2 obtains a pure red component and method 1 obtains more colour variations around the reddish pigment. The proposed methods can provide additional tools for the objective, non-contact analysis of the pigments used in artworks. In this application case, Method 1 will be selected by curators; thus, all of the red pigment is not from the original painting and needs to be eliminated.

## 6. Patents

Patent ES2819052-B2. Cámara multi o hiperespectral para mantener el aumento lateral ajustando el enfoque. Fernández-Balbuena A. A., Bernárdez Vilaboa, R., Molini, D. V., Pinilla, S. M., & Gómez Manzanares, A.

Patent ES2911099-B2. Instrumento y método para calibrar la uniformidad de la iluminación con aplicación en medida de reflectancia con imágenes multiespectrales o hiperespectrales. Fernández-Balbuena A. A., Gómez Manzanares, A., Molini, D. V., Pinilla, S. M., Martínez-Antón, J. C., & Bernárdez Vilaboa, R.

## Figures and Tables

**Table 1:** Results obtained with Methods 1 and 2.

Segmentation Area		Amount of the Segmentation Area Affected by Reddish Pigment					
		Method 1			Method 2		
Pixels	Area (cm^2^)	Pixels	Area (cm^2^)	Percentage of Segmentation Area	Pixels	Area (cm^2^)	Percentage of Segmentation Area
23,829	82.98	7611	26.49	32%	3304	11.5	13.87%

## Data Availability

Data are available on request from the corresponding author, due to restrictions on museum data related to Dalí’s painting.
